# Effect of COVID-19 on childhood *Mycoplasma pneumoniae* infection in Chengdu, China

**DOI:** 10.1186/s12887-021-02679-z

**Published:** 2021-04-28

**Authors:** Ying Zhang, Yijie Huang, Tao Ai, Jun Luo, Hanmin Liu

**Affiliations:** 1 Key Laboratory of Birth Defects and Related Diseases of Women and Children (Sichuan University), West China Second University Hospital, Ministry of Education, Sichuan University, No.20 section3 South Renmin Road, Sichuan Province 610041 Chengdu, China; 2 Children respiratory department,Chengdu Women’s and Children’s Central Hospital, School of Medicine, University of Electronic Science and Technology of China, 611731 Chengdu, China

**Keywords:** *Mycoplasma pneumoniae*, COVID-19 pandemic, Children, Western China

## Abstract

**Background:**

Following the outbreak of the COVID-19 pandemic, a change in the incidence and transmission of respiratory pathogens was observed. Here, we retrospectively analyzed the impact of COVID-19 on the epidemiologic characteristics of *Mycoplasma pneumoniae* infection among children in Chengdu, one of the largest cities of western China.

**Method:**

*M. pneumoniae* infection was diagnosed in 33,345 pediatric patients with respiratory symptoms at the Chengdu Women’s & Children’s Central Hospital between January 2017 and December 2020, based on a serum antibody titer of ≥1:160 measured by the passive agglutination assay. Differences in infection rates were examined by sex, age, and temporal distribution.

**Results:**

Two epidemic outbreaks occurred between October-December 2017 and April-December 2019, and two infection peaks were detected in the second and fourth quarters of 2017, 2018, and 2019. Due to the public health response to COVID-19, the number of positive *M. pneumoniae* cases significantly decreased in the second quarter of 2020. The number of *M. pneumoniae* infection among children aged 3–6 years was higher than that in other age groups.

**Conclusions:**

Preschool children are more susceptible to *M. pneumoniae* infection and close contact appears to be the predominant factor favoring pathogen transmission. The public health response to COVID-19 can effectively control the transmission of *M. pneumoniae*.

## Background

*Mycoplasma pneumoniae* (*M. pneumoniae*) is one of the most common pathogens of respiratory infections in children and adolescents, accounting for up to 40 % of community-acquired pneumonia (CAP) in children over 5 years of age [1], and this percentage rises during epidemics. In most cases, *M. pneumoniae* infections are self-limiting, but they can cause refractory pneumonia and extrapulmonary injuries, leading to severe complications and even death. The growing severity of this disease [2–4] and the occurrence of *M. pneumoniae* epidemics [5] have been associated with macrolide resistance [6–10], which is much higher in Asia than in Europe and North America due to the unregulated use of antibiotics.

Since the first COVID-19 outbreaks in Wuhan, China, in December 2019, the Chinese government responded rapidly and effectively to control the pandemic with restrictive measures that significantly affected the transmission of other respiratory pathogens, including *M. pneumoniae*. In this study, we conducted a retrospective epidemiologic analysis of data from January 2017 to December 2020 in order to evaluate the impact of the public health response to COVID-19 on the epidemiological characteristics and transmission of *M. pneumoniae* among children in western China.

## Method and materials

### Study subjects

Data were retrospectively analyzed for children between 1 month and 18 years of age who came to Chengdu Women’s & Children’s Central Hospital from January 2017 to September 2020 due to respiratory symptoms. The patients’ demographic features, clinical information, and laboratory data were retrospectively collected from the hospital records. The pediatric patients were divided into four groups depending on their age in years: 0–2, 3–6, 7–12, and 13–18.

### Detection of M. pneumoniae

Serum antibodies against *M. pneumoniae* in serum were detected using a passive agglutination kit (Fujirebio, Japan) based on the manufacturer’s instructions. A single titer of ≥1:160 was considered an indicator of *M. pneumoniae* infection.

### Statistical analysis

All data were analyzed using the SPSS software package (version 20.0, IBM, USA). Categorical data were reported as ratios or n (%).

## Results

### Demographic characteristics of pediatric patients with ***M. pneumoniae*** infection

**Fig. 1:**
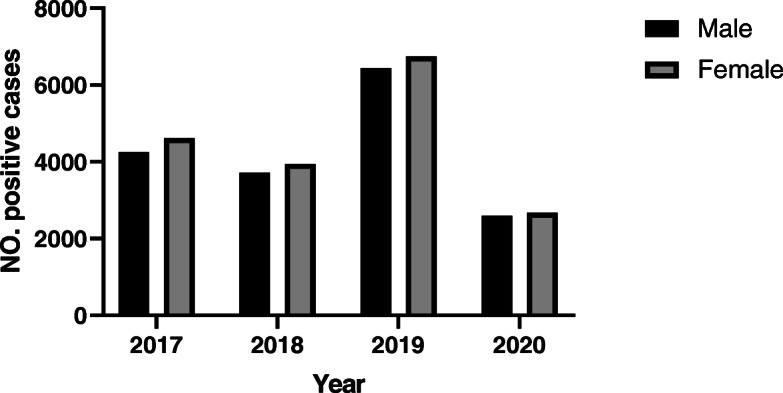
Sex distribution of pediatric patients with *Mycoplasma pneumoniae* infection between January 2017 and December 2020

**Fig. 2:**
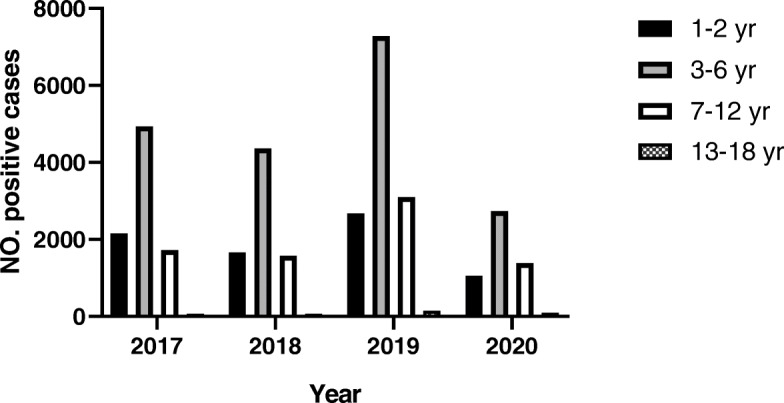
Age distribution of pediatric patients with *Mycoplasma pneumoniae* infection between January 2017 and December 2020

A total of 34,977 pediatric patients were enrolled in the study, including 17,005 males and 17,972 females. The male/female ratios were 0.92:1 for 2017, 0.94:1 for 2018, 0.96:1 for 2019, and 0.97 for 2020 (Fig. 1). In each year, the number of *M. pneumoniae* infection was higher for the age group of 3–6 years than for other age groups, especially in 2019 (Fig. 2).

### Temporal distribution of pediatric patients with ***M. pneumoniae*** infection

**Fig. 3:**
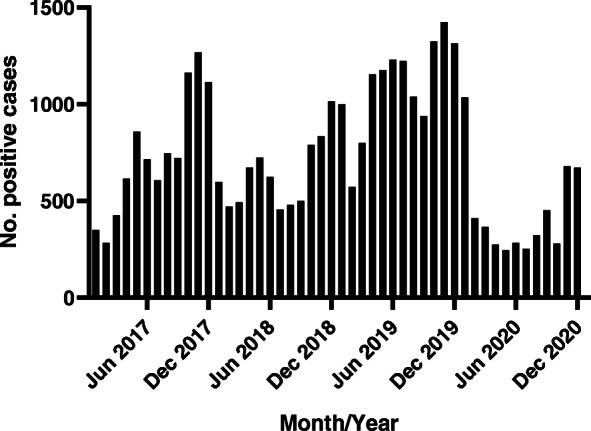
Monthly of pediatric patients with *Mycoplasma pneumoniae* infection between January 2017 and December 2020

**Fig. 4:**
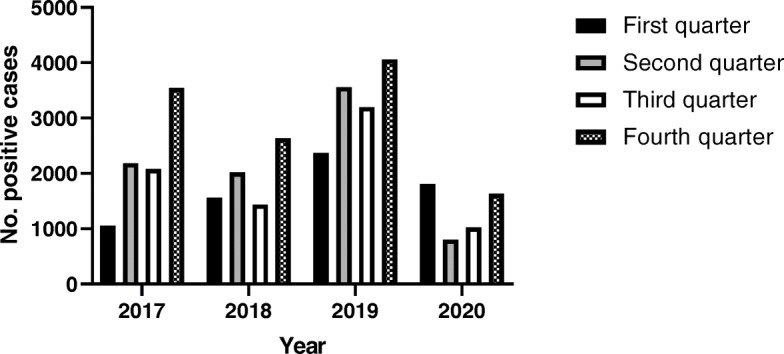
Quarterly distribution of pediatric patients with *Mycoplasma pneumoniae* infection between January 2017 and December 2020

Our data provide the first evidence that two *M. pneumoniae* epidemic outbreaks occurred in western China between 2017 and 2020; the first between October 2017 and December 2017, and the second between April 2019 and January 2020. Analysis of the monthly distribution in the indicated period revealed that the number of *M. pneumoniae* positive cases was the highest in January 2020 and decreased sharply after February 2020 (Fig. 3). In addition, two epidemic peaks were identified in the second and fourth quarters of 2017, 2018 and 2019 (Fig. 4). Interestingly, these peaks decreased significantly after the COVID-19 pandemic outbreak, especially during the second quarter of 2020 (Fig. 4).

### Inpatient/outpatient ratio of pediatric patients with ***M. Pneumoniae*** infection

**Fig. 5:**
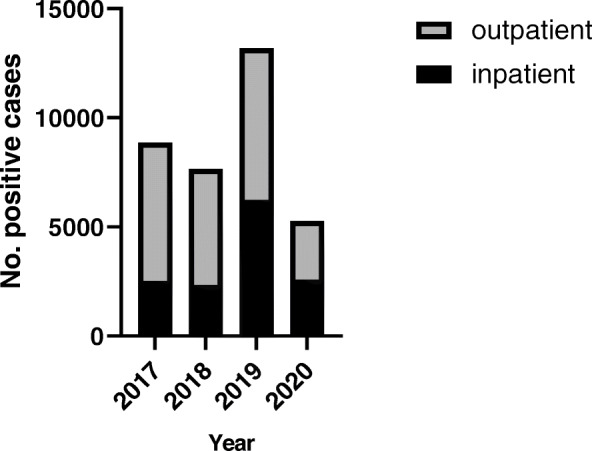
Populations of inpatients and outpatients among pediatric cases of *Mycoplasma pneumoniae* infection between January 2017 and December 2020

The annual hospitalization rates between 2017 and 2020 were 28.5 %, 30.7 %, 47.3 %, and 49.0 %. The highest absolute total number of pediatric patients with *M. pneumoniae* infection, including both outpatients and inpatients, was observed in 2019. The number of inpatients was much higher in 2019–2020 than in 2017–2018. In contrast, the total number of positive cases was significantly reduced in 2020, but the inpatient/outpatient ratio remained almost the same as in 2019 (Fig. 5).

## Discussion

Although epidemiological studies on *M. pneumoniae* infection have indicated that epidemics usually occur every 3–5 years [11, 12], infection incidence in Europe and Asia significantly increased in 2011–2012, 2015, and 2017 [2, 9, 11, 13–15]. Our study retrospectively analyzed the impact of the public health response to COVID-19 on the occurrence of *M. pneumoniae* infection among children in western China, based on analysis of data from 2017 to 2020. In particular, we found that a small-scale epidemic outbreak of 3 months occurred in 2017, while a large-scale outbreak of 10 months occurred in 2019, confirming the uniform global epidemic pattern of *M. pneumoniae* infection. It has also been reported that a long epidemic affecting a large area can lead to a secondary peak in the same epidemic [16]. The average number of *M. pneumoniae* infections per month was approximately two times higher during each epidemic than between the epidemics.

Although substantial numbers of children were diagnosed with *M. pneumoniae* infection throughout the study period, the epidemic peaked in the fourth quarter of each year between 2017 and 2019, which was consistent with the results obtained previously in South Korea [2, 17], USA [3], Israel, and 11 countries of Europe [16]. However, the tendencies in these studies differ from the data reported in epidemiological studies in Italy [18], South Africa [19], and other regions of China [8, 20, 21]. The peaks of *M. pneumoniae* infection between 2017 and 2019 in our study coincided with the school semesters, and the number of infections fell significantly after schools were closed to limit the COVID-19 pandemic. These results indicate that closed settings with closer contacts promoted the *M. pneumoniae* transmission, consistent with studies reporting that *M. pneumoniae* infections are transmitted mainly through droplets spread during close contact [22], and that closed or semi-closed communities, such as military bases, hospitals, religious communities, schools, and institutions are areas associated with the highest rates of transmission, which can more easily lead to epidemics [10, 23–25].

It has also been reported that climate conditions, such as humidity and temperature, can significantly affect the survival and spread of airborne *M. pneumoniae* [26, 27]. However, these studies have come to conflicting conclusions, suggesting that climatic factors are not the primary determinants of *M. pneumoniae* transmission patterns.

Furthermore, no clear differences were observed in sex distribution of pediatric cases of *M. pneumoniae* infection, but the number of positive cases varied significantly depending on age. Some studies have shown that *M. pneumoniae* infections are more common in children over 5 years of age [12], although they also occur in infants [11, 18, 28]. However, other studies have variably suggested higher rates of infection among preschool children or among school-age children [2, 8, 14, 29, 30]. In the present study, the highest number of infections was detected among preschool children especially 3–6 years old, who spent most of the day playing with other children in their same age group in community or daycare settings, where inter-child contact was closer than in primary and secondary schools, thus favoring the transmission of *M. pneumoniae*.

In the present study, the rate of hospitalization due to *M. pneumoniae* infection was within the rates reported in recent studies (18–67 %) [13, 17, 25]. The significant increase in 2019 suggests that infections were more severe during an *M. pneumoniae* epidemic. Nevertheless, the incidence of infections decreased significantly in 2020 due to the restrictive measures and strong isolation policy applied from February 2020 by the Chinese government after the COVID-19 outbreak. In fact, the number of *M. pneumoniae* infections in the second quarter of 2020 was 63.3 %, 60.3 %, and 77.5 % smaller, respectively, than the numbers in the second quarter of 2017, 2018, and 2019 in the condition that the number of *M. pneumoniae* infections in the first quarter was higher than 2017 and 2018. This suggests that a comprehensive public health policy which response to the COVID-19 can effectively reduce M. pneumoniae infections in children [24, 25].

Our study had some limitations, including the fact that *M. pneumoniae* infection was diagnosed based only on a single acute-phase serum antibody titer ≥ 1:160 which was tested by passive agglutination. A 4-fold increase in antibody titer and a single titer ≥ 1:640 were most specific for the diagnose of current or recent *M. pneumoniae* infection [12], while RNA or DNA tested by polymerization chain reaction (PCR) was most sensitive [31]. Given antibodies against *M. pneumoniae* could be affected by co-infection and patients’ immune state [31, 32], and results of PCR could be affected by antibiotics, techniques, and asymptomatic carriage [33, 34], a combination of multiplex-PCR and serology helps to reduce each other’s ‘false positive’ and ‘false negative’ rates and was considered to have the highest specificity and sensitivity [31]. However, paired serum samples are difficult to obtain in pediatric, and study demonstrated that titer of 1:160 had a high sensitivity and the highest Youden index and Kappa value using PCR as the standard indicating that it was conducive to screening for *M. pneumoniae* infection [35], coupled with that multiplex-PCR is expensive, therefore, our retrospective study relied mainly on clinical manifestations of respiratory infection combined with a single antibody titer ≥ 1:160. Another, we have to make longer observation and get more systematic surveillance data for better understanding of the epidemiology of *M. pneumoniae* in COVID-19 pandemic.

## Conclusions

In conclusion, we demonstrate that two epidemic outbreaks of *M. pneumoniae* infection occurred during 2017–2020 in western China. Preschool children were more susceptible to infection, and the predominant factor influencing *M. pneumoniae* transmission appeared to be close contact, especially in childcare centers. The significant differences in the temporal distribution and the decrease in the number of positive cases in the first three quarters of 2020 indicated that the public health response to the COVID-19 pandemic may have effectively controlled the transmission of *M. pneumoniae* infection.

## Data Availability

The datasets used and/or analyzed during the current study are available from the corresponding author on reasonable request.

## Abbreviations

*M. pneumoniae*: *Mycoplasma pneumoniae*
COVID-19: Coronavirus disease 2019
CAP: Community-acquired pneumonia
PCR: Polymerization chain reaction

## References

[1] Atkinson TP, Waites KB (2014). Mycoplasma pneumoniae Infections in Childhood. Pediatr Infect Dis J.

[2] Lee E, Kim CH, Lee YJ (2020). Annual and seasonal patterns in etiologies of pediatric community-acquired pneumonia due to respiratory viruses and Mycoplasma pneumoniae requiring hospitalization in South Korea. BMC Infect Dis.

[3] Watkins LKF, Olson D, Diaz MH (2017). Epidemiology and Molecular Characteristics of Mycoplasma pneumoniae During an Outbreak of M. pneumoniae-associated Stevens-Johnson Syndrome. Pediatr Infect Dis J.

[4] Waites KB, Xiao L, Liu Y, Balish MF, Atkinson TP (2017). Mycoplasma pneumoniae from the Respiratory Tract and Beyond. Clin Microbiol Rev.

[5] Qu J, Chen S, Bao F, Gu L, Cao B (2019). Molecular characterization and analysis of Mycoplasma pneumoniae among patients of all ages with community-acquired pneumonia during an epidemic in China. Int J Infect Dis.

[6] Tanaka T, Oishi T, Miyata I (2017). Macrolide-Resistant Mycoplasma pneumoniae Infection, Japan, 2008–2015. Emerg Infect Dis.

[7] Lee H, Yun KW, Lee HJ, Choi EH (2018). Antimicrobial therapy of macrolide-resistant Mycoplasma pneumoniae pneumonia in children. Expert Rev Anti Infect Ther.

[8] Guo DX, Hu WJ, Wei R (2019). Epidemiology and mechanism of drug resistance of Mycoplasma pneumoniae in Beijing, China: A multicenter study. Bosn J Basic Med Sci.

[9] Dumke R, Schnee C, Pletz MW (2015). Mycoplasma pneumoniae and Chlamydia spp. infection in community-acquired pneumonia, Germany, 2011–2012. Emerg Infect Dis.

[10] Diaz MH, Benitez AJ, Winchell JM (2015). Investigations of Mycoplasma pneumoniae infections in the United States: trends in molecular typing and macrolide resistance from 2006 to 2013. J Clin Microbiol.

[11] Kurkela S, Puolakkainen M, Hokynar K, et al. Mycoplasma pneumoniae outbreak, Southeastern Finland, 2017–2018: molecular epidemiology and laboratory diagnostic lessons. *Eur J Clin Microbiol Infect Dis*. 2019; 38(10):1867–1871. DOI:10.1007/s10096-019-03619-7. 10.1007/s10096-019-03619-7 PMC6778538 31263967

[12] Lee KL, Lee CM, Yang TL (2021). Severe Mycoplasma pneumoniae pneumonia requiring intensive care in children, 2010–2019. J Formos Med Assoc.

[13] Qu J, Yang C, Bao F, Chen S, Gu L, Cao B. Epidemiological characterization of respiratory tract infections caused by Mycoplasma pneumoniae during epidemic and post-epidemic periods in North China, from 2011 to 2016. *BMC Infect Dis*. 2018; 18(1):335. Published 2018 Jul 17. DOI:10.1186/s12879-018-3250-2. 10.1186/s12879-018-3250-2 PMC6050680 30016939

[14] Su M, Wang Q, Li D (2021). Prevalence and clinical characteristics of hospitalized children with community-acquired Mycoplasma pneumoniae pneumonia during 2017/2018, Chengde, China. Med (Baltim).

[15] Nakamura Y, Oishi T, Kaneko K (2021). Recent acute reduction in macrolide-resistant Mycoplasma pneumoniae infections among Japanese children. J Infect Chemother.

[16] Beeton ML, Zhang XS, Uldum SA (2020). *Mycoplasma pneumoniae* infections, 11 countries in Europe and Israel, 2011 to 2016. Euro Surveill.

[17] Eun BW, Kim NH, Choi EH, Lee HJ. Mycoplasma pneumoniae in Korean children: the epidemiology of pneumonia over an 18-year period [published correction appears in J Infect. 2011 Oct;63(4):320]. *J Infect*. 2008; 56(5):326–331. DOI:10.1016/j.jinf.2008.02.018. 10.1016/j.jinf.2008.02.018 18420275

[18] Defilippi A, Silvestri M, Tacchella A (2008). Epidemiology and clinical features of Mycoplasma pneumoniae infection in children. Respir Med.

[19] Carrim M, Wolter N, Benitez AJ (2018). Epidemiology and Molecular Identification and Characterization of Mycoplasma pneumoniae, South Africa, 2012–2015. Emerg Infect Dis.

[20] Chen K, Jia R, Li L, Yang C, Shi Y (2015). The aetiology of community associated pneumonia in children in Nanjing, China and aetiological patterns associated with age and season. BMC Public Health.

[21] Jiang Q, Yang F, Peng Y, Dong X, Ge Y (2020). Epidemiology and molecular identification of mycoplasma pneumoniae associated with respiratory infections in Zhejiang province, China, 2008–2017. J Clin Lab Anal.

[22] Steinberg P, White RJ, Fuld SL, Gutekunst RR, Chanock RM, Senterfit LB (1969). Ecology of Mycoplasma pneumoniae infections in marine recruits at Parris Island, South Carolina. Am J Epidemiol.

[23] Suzuki Y, Seto J, Shimotai Y (2019). Polyclonal spread of multiple genotypes of Mycoplasma pneumoniae in semi-closed settings in Yamagata, Japan. J Med Microbiol.

[24] Zhang X, Han MN, Dong JH (2020). Outbreak of Mycoplasma pneumoniae at a military academy. Mil Med Res.

[25] Zhang WZ, Zhang SJ, Wang QY (2019). Outbreak of macrolide-resistant mycoplasma pneumoniae in a primary school in Beijing, China in 2018. BMC Infect Dis.

[26] Wright DN, Bailey GD, Goldberg LJ (1969). Effect of temperature on survival of airborne Mycoplasma pneumoniae. J Bacteriol.

[27] Tian DD, Jiang R, Chen XJ, Ye Q. Meteorological factors on the incidence of MP and RSV pneumonia in children. PLoS One. 2017;12(3):e0173409. DOI:10.1371/journal.pone.0173409. Mar 10. 10.1371/journal.pone.0173409 PMC5345804 28282391

[28] Oumei H, Xuefeng W, Jianping L (2018). Etiology of community-acquired pneumonia in 1500 hospitalized children. J Med Virol.

[29] Chen A, Song L, Chen Z (2019). Immunoglobulin M profile of viral and atypical pathogens among children with community acquired lower respiratory tract infections in Luzhou, China. BMC Pediatr.

[30] Kumar S, Kashyap B, Kumar S, Kapoor S (2020). Diagnostic utility of serology and polymerase chain reaction for detection of *Mycoplasma pneumoniae* and *Chlamydophila pneumoniae* in paediatric community-acquired lower respiratory tract infections. Indian J Med Microbiol.

[31] Wang L, Feng Z, Zhao M, et al. A comparison study between GeXP-based multiplex-PCR and serology assay for Mycoplasma pneumoniae detection in children with community acquired pneumonia. *BMC Infect Dis*. 2017;17(1):518. Published 2017 Jul 25. DOI:10.1186/s12879-017-2614-3. 10.1186/s12879-017-2614-3 PMC5527399 28743259

[32] Kumar S (2018). Mycoplasma pneumoniae: A significant but underrated pathogen in pediatric community-acquired lower respiratory tract infections. Indian J Med Res.

[33] Li J, Sun L, Wu X (2019). Early Diagnosis of *Mycoplasma pneumoniae* in Children: Simultaneous Amplification and Testing (SAT) Is the Key. Front Pediatr.

[34] Jeon HE, Kang HM, Yang EA (2021). Early Confirmation of *Mycoplasma pneumoniae* Infection by Two Short-Term Serologic IgM Examination. Diagnostics (Basel).

[35] Tang M, Wang D, Tong X (2021). Comparison of different detection methods for Mycoplasma pneumoniae infection in children with community-acquired pneumonia. BMC Pediatr.

